# Novel Synthesis of Nitrogen-Containing Bio-Phenol Resin and Its Molten Salt Activation of Porous Carbon for Supercapacitor Electrode

**DOI:** 10.3390/ma12121986

**Published:** 2019-06-20

**Authors:** Tao Ai, Zhe Wang, Haoran Zhang, Fenghua Hong, Xin Yan, Xinhua Su

**Affiliations:** 1 School of Materials Science & Engineering, Chang’an University, Xi’an 710061, China; solarannn@163.com (H.Z.); 2017131041@chd.edu.cn (F.H.); xinyan@chd.edu.cn (X.Y.); suxinghua@chd.edu.cn (X.S.); 2 Engineering Research Center of Pavement Materials, Ministry of Education of P.R. China, Chang’an University, Xi’an 710061, China

**Keywords:** nitrogen-doped, bio-phenol resin, porous carbon, molten salt, supercapacitor, electrode material

## Abstract

Nitrogen hybridization is an attractive way to enhance the wettability and electric conductivity of porous carbon, which increases the capacitance of carbon-based supercapacitor, however, there is lack of low-cost methods to prepare the nitrogen-doped porous carbon materials. Herein, a novel facile nitrogen-containing bio-phenolic resin was synthesized by polymerization of the carbamate bio-oil, Phenol and paraformaldehyde. As a precursor of nitrogen-doped porous carbon, the nitrogen-containing bio-phenol resin was activated by the one-step molten-salt method. The resultant nitrogen-doped porous carbon showed a high specific surface area up to 1401 m^2^·g^−1^. As a supercapacitor electrode, the nitrogen-doped porous carbons showed specific capacitance of 159 F·g^−1^ at 0.5 A·g^−1^. It also exhibited high cyclic stability with 94.8% retention of the initial specific capacitance over 1000 charge-discharge cycles at 1.0 A·g^−1^. The results suggest that these nitrogen-containing bio-phenol resin provide a new source of nitrogen-doped porous carbon for high-performance supercapacitor electrodes.

## 1. Introduction

A supercapacitor is a new generation of energy storage device, and its core component is electrode material. Among the electrode materials, porous carbon materials were the first to be studied and the most mature for technical application [1,2]. The incorporation of nitrogen into the porous carbon structure can improve its wettability, conductivity, and increases its specific capacitance [3]. There are two ways to incorporate the nitrogen element into the porous carbons [4]. One is the treatment of porous carbon by nitrogenous compound at high temperature; The other is carbonization of nitrogen-containing carbon precursors, for example, nitrogen-containing phenolic resins. Because the latter have higher nitrogen content and more stable cycling stability in super capacitors than the former, there is a lot of literature on nitrogen-containing phenolic resins for preparing nitrogen-doped porous carbons [5,6,7,8,9]. However, the preparation of nitrogen-containing phenolic resin is not effective, which hinders the mass production of nitrogen-doped porous carbons.

In recent years, with the development of sustainable chemical technology, the cheap bio-oil, which is generally produced by fast pyrolysis of rich lignocellulosic biomass, can often be used as partial substitute of phenol to synthesize bio-based phenolic resin [10,11,12,13,14,15]. Bio-oil provides a promising renewable resource to substitute petroleum-based phenol; however, few studies have used phenol-rich bio-oil to synthesize nitrogen-containing phenolic resin. 

In this study, we present a novel synthesis of nitrogen-containing phenol resin by amino esterification bio-oil. The nitrogen-containing phenolic resin was facilely synthesized by phenol, formaldehyde and the amino esterification bio-oil, then, the bio-phenol resin as a precursor of nitrogen-doped porous carbon was simultaneously carbonized and activated in molten salt with one-step method. The nitrogen-doped porous carbons show excellent supercapacitor performance.

## 2. Materials and Methods

### 2.1. Chemicals and Materials

Bio-oil was produced from rapid pyrolysis of Poplar Sawdust at 500 °C. Urea, Phenol and Paraformaldehyde were analytical grade and purchased from Shanghai Macklin Biochemical Co., Ltd. (Shanghai, China).

### 2.2. Synthesis of Nitrogen-Containing Bio-Phenolic Resin

The 50 g of bio-oil was added into a 3-neck boiling flask. The flask was heated by an oil bath to 130 °C until melted. An appropriate amount of urea (bio-oil to mass urea ratio of 100:50) was added and stirred evenly then heated to 150 °C and held until no ammonia escaped. After the reaction finished, the products were naturally cooled to room temperature and the black bitumen solid, called carbamate bio-oil, was obtained. A specific amount of carbamate bio-oil was added into the boiling flask, heated by oil bath to 90 °C. Phenol and poly formaldehyde (50% of the quality of carbamate bio-oil) was added. The mixture was held at 90 °C for 5 h. The product became a bio-phenolic resin after cooling. 

### 2.3. Preparation of Nitrogen-Doped Porous Carbon

The bio-phenolic resin was cured at 100 °C for 1 h then at 180 °C for 2 h. The 3 g of heat cured bio-phenolic resin was weighed and mixed with a precise molar ratio of NaCl-KCl-KOH (NaCl:KCl:KOH = 4:4:1) salt. The mixture was placed in a tube furnace and heated from room temperature to 900 °C in a high purity N_2_ atmosphere. The mixture was held at 900 °C for 4 h before being allowed to cool to room temperature. The product was washed with a 0.1 mL/L solution of HCL and Deionized water repeatedly until the filtrate neutralized. The product was then dried at 80 °C for 12 h to form the porous carbon.

### 2.4. Characterization of Resin and Porous Carbon

The molecular structure of the bio-oil after amino esterification was determined by an infrared (IR) spectrometer (Nicolet IS10, Thermo Scientific, Waltham, MA, USA). The thermogravimetric (TG) analysis of the phenolic resin was carried out by the Thermogravimetric Analyzer (Netzsch TG209F1Libra®, Selb, German). The nitrogen content of phenolic resins was characterized by the elemental analyzer (Vario EL cube, Elementar, Langenselbold, German). The morphology of the specimens was determined using emission scanning electron microscopy (SEM) (Hitachi S-4800, Tokyo, Japan). The XRD patterns of specimens were investigated with a powder diffractometer (Bruker D8 Davinci, Leipzig, Germany). Raman spectra were recorded on a Raman spectrometer (JY HR800, Horiba, Montpellier, France). The pore structure of the specimen was determined by Nitrogen adsorption-desorption isotherms at 77 K on an Automatic adsorption instrument (Mike ASAP2460, Micromeritics Instrument Corp., Norcross, GA, USA).

### 2.5. Preparation of Working Electrode and Electrochemical Analysis

The working electrode was prepared as follow: The porous carbon material (80 wt.%), acetylene black (10 wt.%) and polytetrafluoroethylene (10 wt.%) were used to prepare a uniform paste. The paste was then coated on a nickel foam current collector.

The electrochemical analysis of the specimen was carried out using a three-electrode configuration on the electrochemical workstation (CHI CHI660E, Shanghai, China) in a 6 mol/L KOH electrolyte with a range of −1 to 0 V.

To further evaluate the porous carbon as an electrode in symmetrical supercapacitor device, a CR2032 coin-type cell was assembled using the porous carbon as symmetrical electrodes, using a separator with the electrolyte of 6 M KOH solution. The performance of the device in terms of its energy density (E) and power density (P), which can be estimated using the following equations: E = 1/2CU^2^, P = E/Δt, where C represents the specific capacitance based on the galvanostatic charge-discharge results of supercapacitor, while U refers to the potential change within the discharge time Δt.

## 3. Results and Discussion

### 3.1. IR and TG Analysis

**Figure 1:**
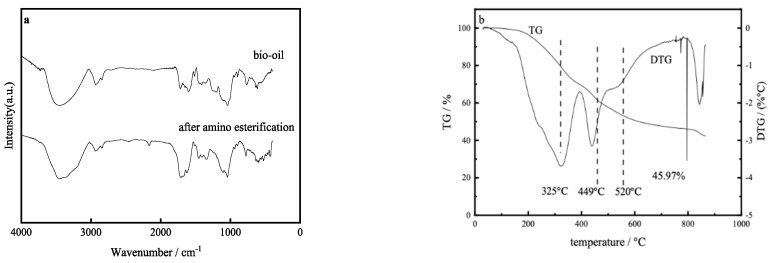
(**a**) IR spectra of bio-oil before and after carbamate; (**b**) TG-DTG curves of bio-phenolic resins.

In order to testify the chemical structure change, carbamate bio-oil was characterized by IR. Figure 1a is the result of IR spectra of carbamate bio-oil. Compared with bio-oil, the carbonyl absorption peak of 1700 cm^−1^ increased noticeably after amino esterification. The 3180–3360 cm^−1^ absorption peak is the symmetric and asymmetric vibration of amino N-H, showing two adjacent strong absorption peaks, while in the bio-oil there is only the absorption peak of hydroxyl in 3300–3500 cm^−1^. Based on the above analysis, it can be seen that carbamate was introduced, there are reactive amides, and the N element was successfully introduced.

Figure 1b is the result of thermogravimetry-derivative thermogravimetry (TG-DTG) curves of bio-phenolic resins. It can be seen that with the amination of bio-oil, the char yield of the bio-phenolic resin is nearly 46% at 800 °C. The higher char yield of the bio-phenolic resin means more resin carbon. These would good for the mass production of nitrogen-doped porous carbons.

### 3.2. SEM and Elemental Analysis

**Figure 2:**
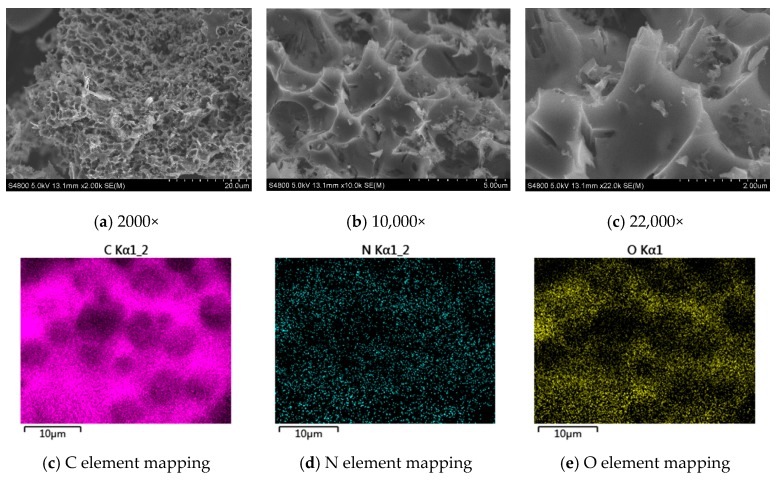
Scanning electron microscopy (SEM) with different magnifications and element mapping images of porous carbon. (**a**) 2000×; (**b**) 10,000×; (**c**) 22,000×; (**d**) C element mapping; (**e**) N element mapping; (**f**) O element mapping.

Molten salt one-step activation is a preparation method for activated porous carbon. Figure 2 is the SEM images of molten salt activated porous carbon specimen derived from bio-phenolic resins. It can be seen that the specimen has an abundant porous structure. Under a high-temperature molten salt environment, a resin precursor can be activated with a KOH activator. In the process of activation, the KOH in the molten salt system reacts with some of the carbon atoms, such as at the edge of the defect structure of the carbon atoms connected with the heteroatoms [16]. This activation makes the pore structure of the carbon material further developed. The microstructure of the activated carbon is changed by the complex carbonization and activation [17] so that it has a microporous and mesoporous structure.

The content of nitrogen in the bio-phenolic resins is important to a property of porous carbon. After testing, the nitrogen content in the bio-phenolic resins was as high as 9%. When these bio-phenolic resins were changed into activated carbon, the nitrogen content of the activated carbon still remains 5.6%. The mapping images of C, N and O elements of the activated carbon are shown in Figure 2. It is known that the elements C, N and O are distributed in large quantities evenly throughout the materials. The result indicates that the N and O elements in the bio-phenolic resin can be retained and the activated carbon is nitrogen-doped porous carbon.

### 3.3. XRD X-ray Diffraction and Raman Spectral Analysis

**Figure 3:**
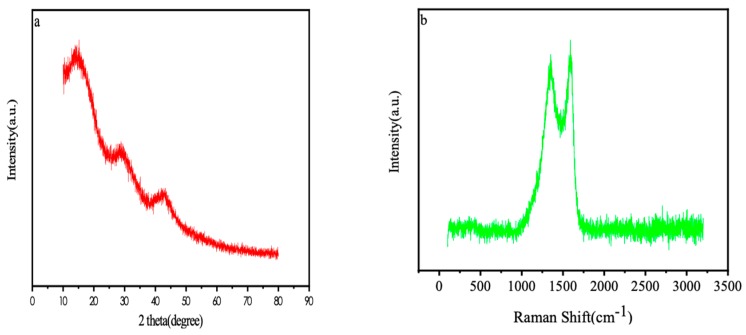
(**a**) XRD patterns of the porous carbon; (**b**) Raman spectrum the porous.

The XRD patterns of the nitrogen-doped porous carbon was shown in Figure 3a. It was showed that the carbon has a broad “steamed bun” diffraction peak at the 28° and 42°. The result represents the existence of the amorphous carbon and graphite structure [18]. Figure 3b is the Raman spectra of the nitrogen-doped porous carbon. It displayed apparent D and G peaks. The D peak is the characteristic absorption peak of amorphous carbon. The G peak is considered as the absorption peak of the graphite structure [19]; therefore, it is confirmed that the nitrogen-doped porous carbon belongs to the amorphous carbon and has a higher degree of graphitization.

### 3.4. Surface Area and Porosity Determination Using N_2_ Adsorption

**Figure 4:**
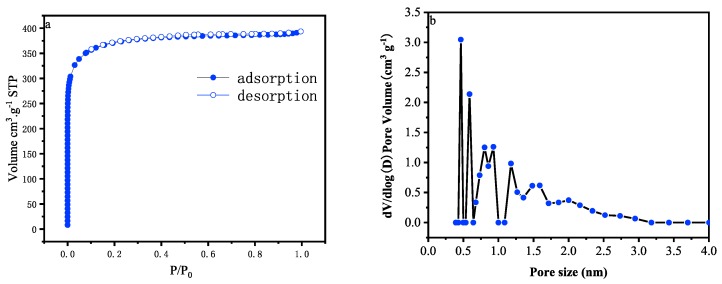
(**a**) Nitrogen adsorption-desorption isotherms; (**b**) pore size distributions.

Figure 4a is the N_2_ absorption-desorption isotherms of the porous carbon. The isotherms show the characteristics of the typical type I isotherms, indicating that the pore distribution is mainly microporous, and has a lower mesoporosity [20,21]. The pore size distribution of the porous carbon is shown in Figure 4b. It is further concluded that the pore size is mainly concentrated below 2 nm. Because of this fact, the activation with KOH produces a large number of micropores, which improves the specific capacitance of the carbon [22], we use this method to produce the porous carbon. The obtained carbon reached the specific surface area and pore volume of 1401 m²/g and 0.61 cm³/g (Table 1), respectively.

### 3.5. Analysis of Electrochemical Energy Storage

**Figure 5:**
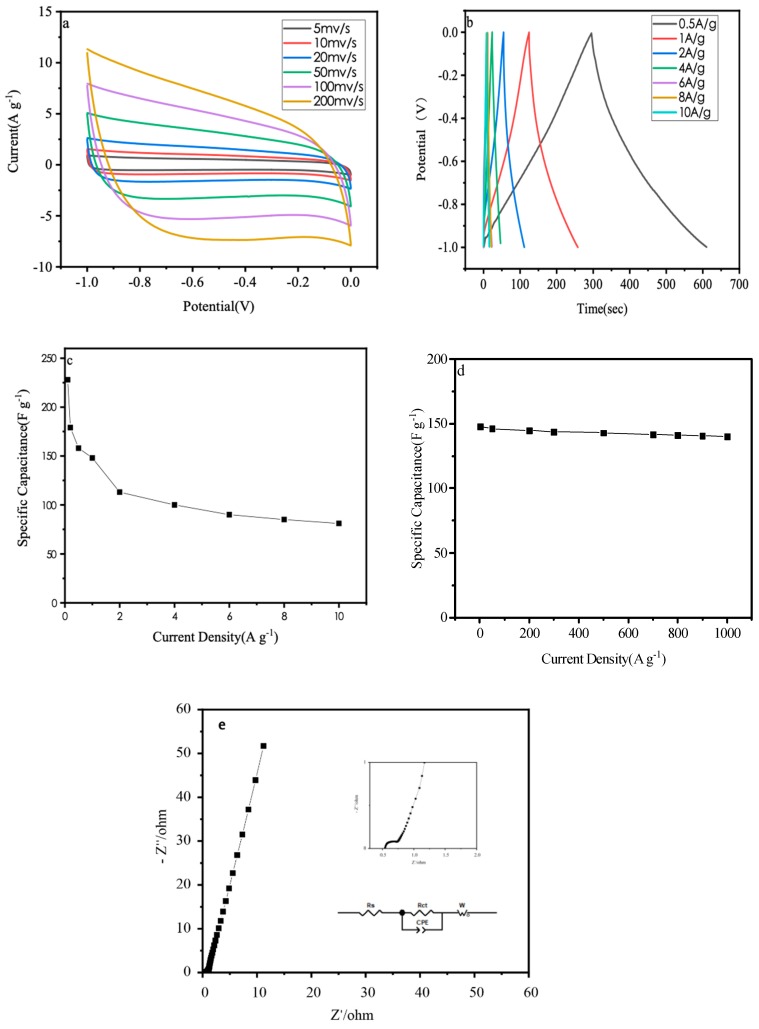
(**a**) cyclic voltammetry (CV) curves of porous carbon at various scan rates; (**b**) galvanostatic charge/discharge curves of porous carbon under various current densities; (**c**) specific capacitance versus current density of porous carbon; (**d**) cycling stability of porous carbon at 1A/g; (**e**) Nyquist plots.

Figure 5a is the cyclic voltammetry (CV) curve of the specimen at different scanning rates. It can be seen that the CV curve of the specimen presents a rectangular shape under cycling rate of 50 mV/s, indicating that the electric double-layer provides most of the capacitance. It also shows that the electrode has better conductivity and higher current response. With the sweep speed increasing to the 200 mv/s, the corresponding curve still has no obvious distortion and keeps the approximate rectangular shape. A gradual increase in the area of the curve shows the good electrochemical characteristics of the material [23].

Figure 5b is a galvanostatic charge/discharge curve with different current densities. The curve approximates an isosceles triangle. Because it shows excellent reversible and charge/discharge performance, this porous carbon can be used as electrode material for a supercapacitor. When the current density is 0.5, 1.0, 2.0, 6.0, 8.0 and 10.0 A/g, the specific capacitance is 159, 148, 113, 90, 85 and 81 F/g respectively. With the increase of current density, the capacitance began to show a certain degree of decline.

Figure 5c is the specific capacitance variation under different current densities. Because the porous structure of specimens is mainly microporous and less mesoporous, when the current density increases from 0.5 A/g to 10 A/g, the specific capacitance drops from 159 F/g to 81 F/g, and the capacitance retention rate is only 52.6%. Mesoporous carbon provides a channel for ion migration. At high current density, ions can migrate quickly in the channel, thus increasing the capacitance retention rate. The low ratio of mesoporous carbon in these specimens lead to unsatisfactory capacitance retention rate [24].

In order to test the cyclic stability of the specimen, 1.0 A/g current density is used to charge/discharge 1000 times. The capacitance retention curve is shown in Figure 5d. After 1000 cycles, the specific capacitance remained of 94.8%. This curve shows that the nitrogen-doped porous carbon electrode has excellent cycling stability [25].

The electrochemical impedance spectroscopy measurement is a useful method to test the conductivity of electrode materials. Measurement results can be shown by Nyquist plot. Figure 5e is the Nyquist plot of the carbon electrodes. It can be seen from the figure that the impedance curve in the high frequency region is a semicircle, reflecting the charge transfer process at the electrode/electrolyte interface. The equivalent impedance simulation analysis is performed on the measured impedance map. As shown in the figure, the RESR value (0.535 Ω) is indicated that the electrode material has a low internal resistance [26].

**Figure 6:**
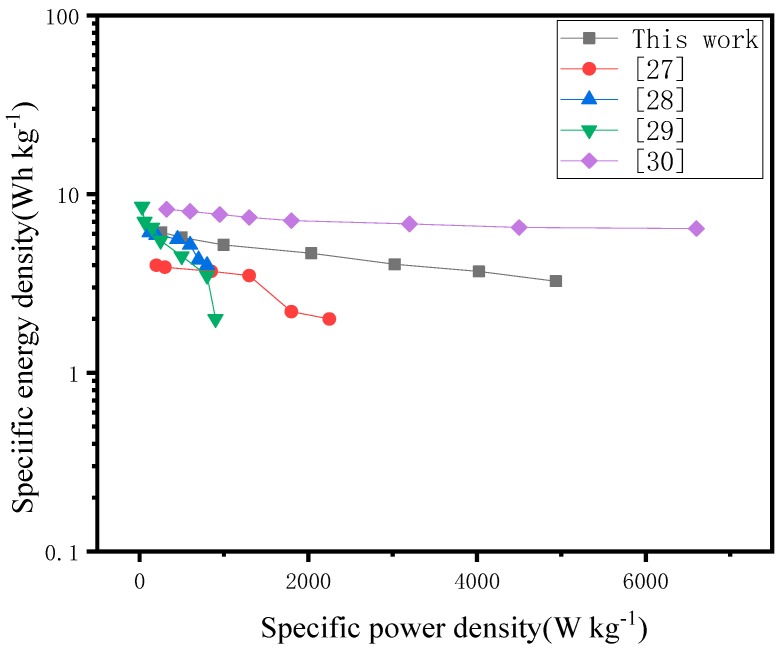
Ragone plot related to energy and power densities of carbon supercapacitor.

A two carbon electrode symmetrical supercapacitor was assembled to evaluate its electrochemical performance. Figure 6 shows the Ragone plot for the symmetrical supercapacitor with the calculated power density and energy density. Compared to other types of activated carbon reported in the literature [27,28,29,30], when the current density is 0.5 A/g, the device shows a good energy density of 6.11 Wh/kg at a power density of 258 W/kg.

## 4. Conclusions

This study provides a novel facile synthesis method of nitrogen-containing bio-phenolic resin. The nitrogen-containing bio-phenolic resin is an ideal precursor for preparation of nitrogen-doped porous carbon. Future work should focus on enlarging the capacity of nitrogen-doped mesoporous carbon. The nitrogen-doped porous carbon electrode has excellent cycling stability and could be used for a wide range of applications in supercapacitor.

## Figures and Tables

**Table 1:** Textural properties of the carbon materials.

S_BET_^a^ (m²/g)	S_micro_^b^ (m²/g)	V_total_^c^ (cm³/g)	V_micro_^d^ (cm³/g)	D ^e^ (nm)
1401	1132	0.609	0.453	1.768

a = Brunauer-Emmett-Teller (BET) surface area. b = Micropore surface area, derived from the t-plot method. c = Total pore volume, measured at P/P0 = 0.98. d Micropore volume, derived from the Dubinin-Astakhov method e Micropore average diameter, calculated by the Barret-Joyner-Halenda (BJH) method.
